# Correlation between patients’ reasons for encounters/health problems and population density in Japan: a systematic review of observational studies coded by the International Classification of Health Problems in Primary Care (ICHPPC) and the International Classification of Primary care (ICPC)

**DOI:** 10.1186/s12875-017-0658-5

**Published:** 2017-09-13

**Authors:** Makoto Kaneko, Ryuichi Ohta, Naoki Nago, Motoharu Fukushi, Masato Matsushima

**Affiliations:** 1 Musashikoganei Clinic, Japanese Health and Welfare Co-operative Federation, 1-15-9, Honcho, Koganei-shi, Tokyo, 184-0004 Japan; 2 Division of Clinical Epidemiology, Jikei University School of Medicine, 3-25-8, Nishishimbashi, Minato-ku, Tokyo, 105-8461 Japan; 3 Unnan City Hospital, Faculty of Community Care, 96-1, Iida, Daito-cho, Unnan city, Shimane 699-1221 Japan; 4 Musashi Kokubunji Park clinic, 2-16-34-127, Nishimoto-machi, Kokubunji-shi, Tokyo, 185-0023 Japan

**Keywords:** Primary care, Reasons for encounters (RFEs), International Classification of Primary Care (ICPC)

## Abstract

**Background:**

The Japanese health care system has yet to establish structured training for primary care physicians; therefore, physicians who received an internal medicine based training program continue to play a principal role in the primary care setting. To promote the development of a more efficient primary health care system, the assessment of its current status in regard to the spectrum of patients’ reasons for encounters (RFEs) and health problems is an important step. Recognizing the proportions of patients’ RFEs and health problems, which are not generally covered by an internist, can provide valuable information to promote the development of a primary care physician-centered system.

**Methods:**

We conducted a systematic review in which we searched six databases (PubMed, the Cochrane Library, Google Scholar, Ichushi-Web, JDreamIII and CiNii) for observational studies in Japan coded by International Classification of Health Problems in Primary Care (ICHPPC) and International Classification of Primary Care (ICPC) up to March 2015.

We employed population density as index of accessibility. We calculated Spearman’s rank correlation coefficient to examine the correlation between the proportion of “non-internal medicine-related” RFEs and health problems in each study area in consideration of the population density.

**Results:**

We found 17 studies with diverse designs and settings. Among these studies, “non-internal medicine-related” RFEs, which was not thought to be covered by internists, ranged from about 4% to 40%. In addition, “non-internal medicine-related” health problems ranged from about 10% to 40%. However, no significant correlation was found between population density and the proportion of “non-internal medicine-related” RFEs and health problems.

**Conclusions:**

This is the first systematic review on RFEs and health problems coded by ICHPPC and ICPC undertaken to reveal the diversity of health problems in Japanese primary care. These results suggest that primary care physicians in some rural areas of Japan need to be able to deal with “non-internal-medicine-related” RFEs and health problems, and that curriculum including practical non-internal medicine-related training is likely to be important.

**Electronic supplementary material:**

The online version of this article (10.1186/s12875-017-0658-5) contains supplementary material, which is available to authorized users.

## Background

In 2013, the Ministry of Health, Labour and Welfare highlighted the importance of the primary care physician in the rapidly aging society of Japan [1]. However, in Japan, a boundary between primary care and secondary care is ambiguous due to free-access system [2] and there are many specialist clinics (e.g., ophthalmological clinic) which deal with health problems of each discipline [3]. In addition, the Japanese health care system has yet to establish structured training for primary care physicians; therefore, physicians who received an internal medicine based training program continue to play a principal role in the primary care setting [3].

To promote the development of a more efficient primary health care system, the assessment of its current status in regard to the spectrum of patients’ reasons for encounters (RFEs) and health problems is an important step [4]. However, the International Classification of Diseases 10th revision (ICD-10) does not include a classification for RFEs or the health problems of unsolved problems [5, 6], which makes it inconsistent with the primary care setting. Therefore, the International Classification of Primary Care 2nd edition (ICPC-2) is recommended for use in primary care[7]^.^ Although several descriptive surveys utilizing ICPC-2 have been conducted in Japan [4], there has been no systematic review which target surveys using ICPC-2. For example, recognizing the proportions of patients’ RFEs and health problems, which are not generally covered by an internist, can provide valuable information to promote the development of a primary care physician-centered system. In addition, Miyazaki presumed that the less accessibility patients have to a specialist clinic and/or a secondary care hospital, the more diverse their RFEs and health problems in the primary care setting [4].

This study aimed to describe the diversity of RFEs and health problems in Japanese settings of primary care. We also examined a correlation between the proportions of non-internal medicine related RFEs/health problems and population density as index of accessibility.

## Methods

### Study design

Systematic review.

### Search strategy

In the present study, we followed the preferred reporting items for systematic reviews and meta-analyses (PRISMA) statement [8]. We searched six databases (PubMed, the Cochrane Library, Google Scholar, Ichushi-Web, JDreamIII and CiNii) for studies in Japan coded by ICHPPC and ICPC up to March 2015. The search strategy was based on the following title/abstract keywords in English and Japanese: (“ICPC” OR “ICPC-2” OR “ICHPPC” OR “International Classification of Primary Care” OR “International Classification of Primary Care-2” OR “International Classification of Health problem in Primary Care”) AND (“Japan”). We also reviewed the reference lists of relevant studies to identify research that might have been missed in the database search.

Ichushi-Web is an online Japanese literature searching system provided by the non-profit Japan Medical Abstracts Society. Ichushi-Web covers about 10 million medical papers from 6000 journals in Japan, and is often used for Japanese literature searches [9].

JDreamIII (Japan Science and Technology Agency Document Retrieval System for Academic and Medical Fields) is an online Japanese literature searching system provided by the Japan Science and Technology Agency. JDreamIII covers about 60 million articles, including serial publications, reports, conference material, public documents and proceedings on science and technology [10].

CiNii is an online Japanese literature searching system provided by the National Institute of informatics. CiNii covers about 18 million articles focusing on natural and cultural science [11].

### Inclusion and exclusion criteria

Literature searches and data extraction were independently conducted by two investigators (M.K. and R.O.), and any discrepancies were resolved by discussion. In the present study, databases were searched for observational studies in Japan coded by ICHPPC, ICHPPC-2, ICHPPC-2-Defined, ICPC and ICPC-2 classifications to evaluate the correlation between patients’ RFEs and health problems and population density. Studies conducted in the hospital setting were excluded because the aim of the study was to clarify the spectrum of RFEs and health problems in primary care. Details of the inclusion criteria are shown in Table 1.

**Table 1:** Inclusion criteria

Study design	Observational study
Date of publication	Until March 31, 2015
Setting	Japan
	Clinic only (Hospital were excluded)
Methods	Coding RFEs or health problems using ICHPPC, ICHPPC-2,
	ICHPPC-2-Defined, ICPC and ICPC-2
Results	Frequency of RFEs and health problems

ICHPPC: International Classification of Health Problems in Primary Care

ICPC: International Classification of Primary Care

RFEs: reasons for encounters

The present study included the following classifications developed by the World Organization of National Colleges, Academies and Academic Associations of General Practitioners/Family Physicians (WONCA) [6]:

ICHPPC: Developed in 1975 to classify health problems in primary care. The classification was mapped to ICD-8.

ICHPPC-2: Developed in 1979 and mapped to ICD-9.

ICHPPC-2-Defined: Developed in 1983. Explanatory remarks were added with ICHPPC-2 to improve usability.

ICPC: Developed in 1987 to combine “Reasons for Encounter Classification (RFEC)” and “International Process in Primary Care (IC-Process-PC)” with the ICHPPC. The classification contained RFEs, including feelings of patients and interventions. The classification was mapped to ICD-10.

ICPC-2: Developed in 1998 and mapped to ICD-10. Explanatory remarks were added with ICPC. This classification is frequently used in primary care settings all over the world. It has been translated into 22 languages.

Studies that did not mention the frequency of RFEs and health problems, studies conducted in countries other than Japan, unpublished data, conference presentations, and conference minutes were all excluded from the present study.

### Data extraction

Extracted information is shown in Table 2. In Japan, patients who have “non-internal medicine-related” RFEs tend to visit specialists as opposed to internists [3]. Therefore, a high percentage of “non-internal medicine-related” RFEs and health problems is thought to indicate the comprehensiveness of RFEs and health problems by the primary care physician. To clarify the comprehensiveness of RFEs in Japanese primary care settings, we calculated the proportions of“non-internal medicine-related RFEs” and “non-internal medicine-related health problems” among the top 20 RFEs and health problems in each study because most of included studies did not report the rank of RFEs and health problems more than the top 20.

**Table 2:** Data extraction

Data	Remarks
Year of publication	
Author	
Setting	The categories of setting are based on description in each included study
Study period	
Number of facilities	
Total number of patients	
Total number of encounters	
Total number of RFEs	
Total number of health problems	
Proportion of “non-internal medicine-related” RFEs in the top 20 RFEs	
Proportion of “non-internal medicine-related” health problems in the top 20 health problems	
Classification	ICHPPC/ICHPPC/ICHPPC-2-Defined
	ICPC/ICPC2
Primary outcome measures	RFEs (first visit, periodic visit)/health problems (acute, chronic)
Distinction between acute and chronic	
Quality of coding	Prospective or retrospective
	Single or multiple evaluator
	Description of coding training
Prospective or retrospective	
Number of evaluators	

RFEs: reasons for encounters

ICHPPC: International Classification of Health Problems in Primary Care

ICPC: International Classification of Primary Care

*Eighteen categories of health problems in the ICHPPC*: Among these categories, “I: Infective and parasitic,” “II: Neoplasms,” “III: Endocrine, nutritional and metabolic” “IV: Blood disease” “VI: Nervous system and sense organs,” “VII: Circulatory system,” “VII: Respiratory system,” “IX: Digestive system,” were defined as “internal medicine-related”. In contrast, “V: Mental disorder,” “X: Genitourinary system(including breast),” “XI: Pregnancy, childbirth and puerperium,” “XII: Skin and subcutaneous tissue,” “XIII: Musculoskeletal and connective tissue,” “XIV: Congenital anomalies,” “XV: Perinatal morbidity,” “XVII: Injuries and adverse effects,” were defined as “non-internal medicine-related”. (“XVI: Signs, symptoms and ill-defined conditions,” and “XVII: Supplementary” were excluded.)

*Seventeen categories of RFEs* and *health problems* in *the ICPC* (Table 3): Among these categories, “A: General and unspecified,” “B: Blood. Blood-forming organs and immune mechanism,” “D: Digestive,” “K: Cardiovascular,” “N: Neurological,” “R: Respiratory” and “T: Endocrine/Metabolic and Nutritional” were defined as “internal medicine-related”. In contrast, “F: Eye,” “H: Ear,” “L: Musculoskeletal,” “P: Psychological,” “S: Skin,” “U: Urological” “W: Pregnancy, Childbearing, Family Planning,” “X: Female genital,” “Y: Male genital” and “Z: Social problems” were defined as “non-internal medicine-related”.

**Table 3:** Examples of ICPC

Category	Example
A: General and unspecified	A01 Pain general/A02 Chill/A03 Fever
B: Blood. Blood-forming organs and immune mechanism	B02 Lymph gland/B04 Blood symptom/B25 Fear of AIDS
D: Digestive	D01 Abdominal pain/D02 Abdominal pain epigastric/D03 Heartburn
F: Eye	F01 Eye pain/F02 Red eyes/F03 Eye discharge
H: Ear	H01 Ear pain/H02 Hearing complaint/H03 Tinnitus
K: Cardiovascular	K01 Heart pain/K02 Pressure/K03 Cardiovascular pain
L: Musculoskeletal	L01 Neck symptom/L02 Back symptom/L03 Low back symptom
N: Neurological	N01 Headache/N02 Face pain/N04 Restless legs
P: Psychological	P01 Feeling anxious/P02 Acute stress reaction/P03 Feeling depressed
R: Respiratory	R01 Pain respiratory system/R02 Shortness of breath/R03 Wheezing
S: Skin	S01 Pain of skin/S02 Pruritus/S03 Warts
T: Endocrine/Metabolic and Nutritional	T01 Excessive thirst/T02 Excessive appetite/T03 Loss of appetite
U: Urological	U01 Dysuria/U02 Urinary frequency/U04 Incontinence Urine
W: Pregnancy, Childbearing, Family planning	W01 Question of pregnancy/W02 Fear of pregnancy/W03 Antepartum bleeding
X: Female genital	X01 Genital pain female/X02 Menstrual period/X03 Intermenstrual pain
Y: Male genital	Y01 Pain penis/Y02 Pain in testis/Y03 Urethral discharge
Z: Social problems	Z01 Poverty/Z02 Food or water problem/Z03 Housing problem

ICPC: International Classification of Primary Care

We were not able to find the definition on the distinction between “internal medicine-related” and “non-internal medicine-related” in the previous reports. Therefore, two of authors, (MM and MK), a Fellow of the Japanese Society of Internal Medicine and a Japan Primary Care Association certified family physician, discussed and defined this distinction for the study. In detail, we discussed which clinical speciality was mainly chosen by patients having the RFEs of each ICPC/ICHPPC- chapter under the situation that both internists and other specialists such as ophthalmologists were equally available. Also, we took into consideration whether an internist referred a patient to specialists.

### Statistical analysis

Usual indicators of accessibility such as “Provider-to-population ratios”, “Travel impedance to nearest provider” and “Average travel impedance to provider” [12] could not be evaluated from the studies conducted in the past and past census data. That was the reason why we employed population density as an index of accessibility, because population density can be used as an indicator of rurality [13]. We then calculated Spearman’s rank correlation coefficient to examine the correlation between the proportion of “non-internal medicine-related” RFEs and health problems in each study area in consideration of the population density.

We calculated the population density in each study based on census data in the administrative district area from the year closest to the study period [14, 15].

## Results

**Fig. 1:**
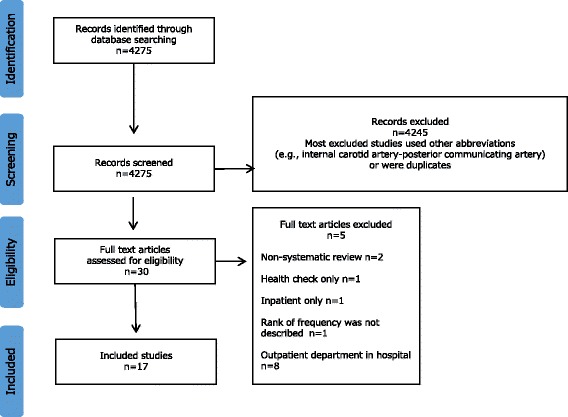
Flow diagram for the selection of studies in the systematic review

After searching through the titles and abstracts of 4275 publications, 17 eligible publications were identified (Fig. 1). Details about the included studies [16–32] are shown in Additional file 1: Table S1 and the characteristics of those studies are shown in Table 4.

**Table 4:** Characteristics of included studies

	Total number (proportion %)
Eligible publications	17
Classification	
ICHPPC	1 (5.9)
ICHPPC-2-Defined	9 (52.9)
ICPC	5 (29.4)
ICPC-2	2 (11.8)
Study period	
< 1 year	4 (23.5)
1 year	10 (58.8)
> 1 year	2 (11.7)
Others: one study contained two study periods (2 years and 4 month)	1 (5.9)
Setting	
Urban clinic	1 (5.9)
Rural clinic	13 (76.5)
Comparison between	1 (76.5)
rural clinic, rural hospital and urban clinic	1 (5.9)
University hospital’s affiliated primary care clinic	1 (5.9)
Number of facilities	
Single	12 (70.6)
Multiple	5 (29.4)
Study design	
Prospective	13 (76.5)
Retrospective	2 (15.4)
Prospective and retrospective	1 (5.9)
No description about study design	1 (5.9)
Number of evaluators	
1 person	11 (64.7)
≥ 2 persons	3 (17.6)
No description about number of evaluators	3 (17.6)
Quality of coding	
There are descriptions about quality of coding	2 (11.8)
There are no descriptions about quality of coding	15 (88.2)
Primary outcome measure	
Only RFEs	1 (5.9)
Only health problems	9 (52.9)
RFEs and health problems	7 (41.2)

ICHPPC: International Classification of Health Problems in Primary Care

ICPC: International Classification of Primary Care

RFEs: reasons for encounter

In these studies, “non-internal medicine-related” RFEs, which was not thought to be covered by internists, varied from approximately 4% to 40%. In addition, “non-internal medicine-related” health problems varied from 10% to 40%. The proportion of “non-internal medicine-related” RFEs reached 41.4% in a study set in rural area. Moreover, the proportion of “non-internal medicine-related” health problems reached 45.4% in another study of rural setting.

**Fig. 2:**
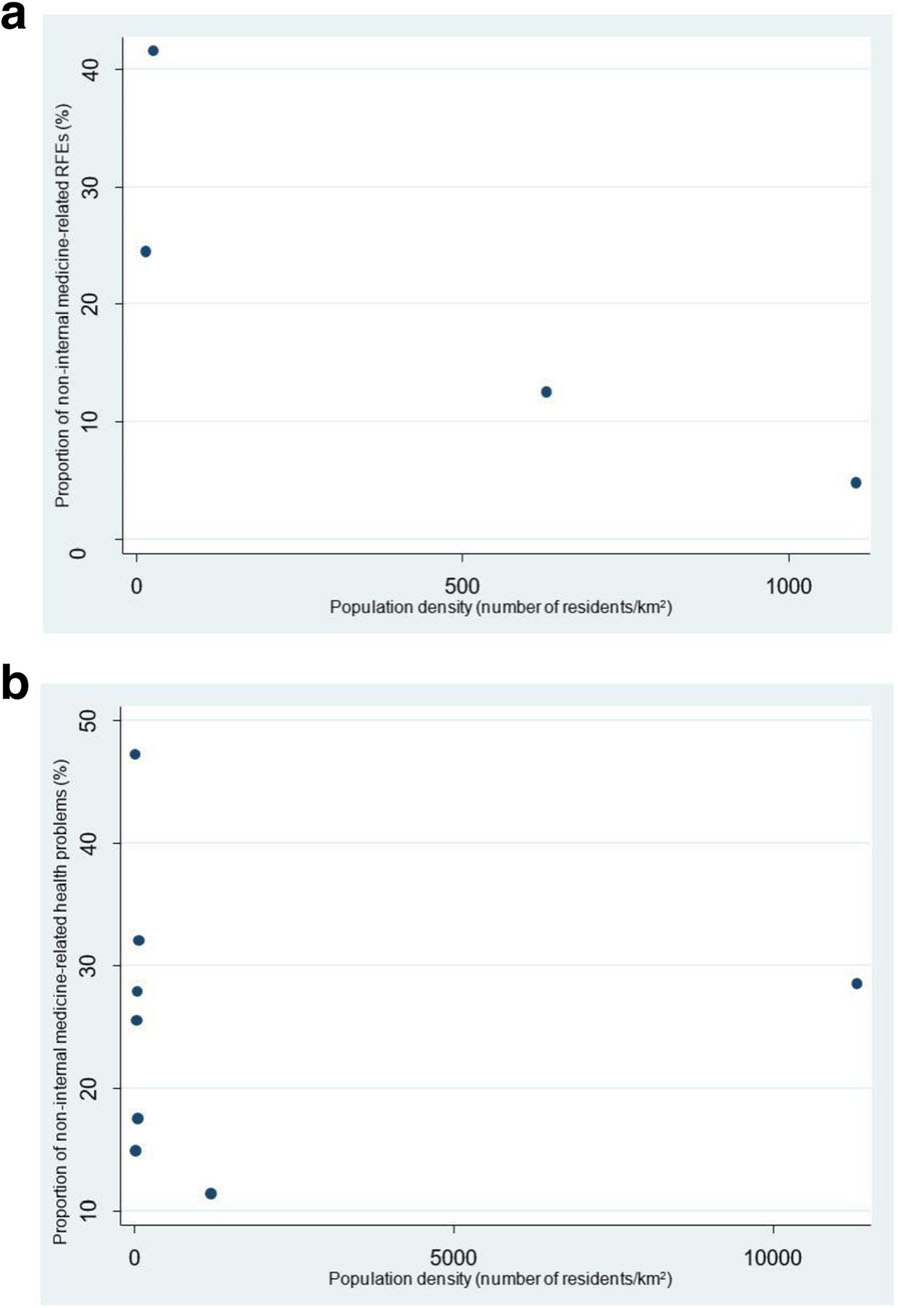
**a** Proportion of “non-internal medicine-related” reasons for encounter (RFEs) among the top 20 RFEs and population density. We used “RFEs in the first visit” for the Figure because most studies described only RFEs in the first visit. **b** Proportion of “non-internal medicine-related” health problems among the top 20 health problems and population density. We included 8 studies in which all health problems (all health problems: combination of acute and chronic health problems) had been described

The relationship between the proportion of “non-internal-medicine-related” RFEs among all RFEs and population density is shown in Fig. 2a, while that between “non-internal-medicine-related” health problems among all health problems and population density is shown in Fig. 2b. We used “RFEs in the first visit” for Fig. 2a and statistical analysis because most studies described only RFEs in the first visit. Five studies in which all health problems (all health problems: combination of acute and chronic health problems) had not been described were excluded from Figure2b and statistical analysis. We used Spearman’s rank correlation coefficient to investigate for the presence of a dependence between RFEs/health problems and population density. In Fig. 2a, there seems to be negative correlation between proportion of non-internal medicine related RFEs and population density. However, no statistically significant correlations were found (*p* = 0.20): Spearman’s rank correlation coefficient was −0.80 (95% Confidence Interval: −0.998 to 0.507). Also, the correlation between health problems and population density was not statistically significant (*p* = 0.74): Spearman’s rank correlation coefficient was −0.14 (95% Confidence Interval: −0.678 to 0.729).

## Discussion

The current systematic review detected 17 studies coded by ICHPPC and ICPC in Japan with diversity in both study design and settings.

In Japan, even in the primary-care setting, the proportion of “non-internal medicine-related” RFEs varies tremendously. In contrast, in other countries with an advanced primary care system, primary care physicians manage more “non-internal medicine-related” RFEs and health problems [33]. The differences between these countries and Japan in the proportion of RFEs are likely due to the Japanese free access system. In Japan, a patient can visit a specialist freely as a first encounter. A primary-care clinic located near a facility with a specialist does not need to manage “non-internal medicine-related” RFEs and health problems. For example, one of the included study [24] in urban area described the non-internal medicine related health problems were only 11.4%. However, in some studies in rural area, the percentage of “non-internal medicine-related” RFEs or health problems reached over 40% [20, 22, 25, 31]. Therefore, to refine and improve the educational system for aspiring generalists in Japan, curriculum with “non-internal medicine-related” practical training should be emphasized.

The shortage of studies in urban or suburban areas may partly explain the lack of a significant correlation between “non-internal medicine-related” RFEs/health problems and population density. For instance, most of the studies (13/17) using ICHPPC and ICPC were carried out in rural areas. In contrast, only two of the studies were conducted in an urban setting. In addition, only 2 studies described the evaluator’s experience of attending ICPC-coding training program, though the quality of data collection is said to be important when using data from patient records such as ICPC [34]. Japanese primary care physicians might obtain more-precise picture by considering surveys in diverse areas and quality of methodology.

It is also important to note that far fewer Japanese studies use ICPC data for purposes such as health care policy and medical education compared with other countries using ICPC data [32, 35–38]. For example, in the Netherlands, more than 300 studies have been conducted from the existing ICPC database [39].

The current study has some limitations. First, population density could only be measured retrospectively. It was not possible to consider detailed medical circumstances such as the existence of a nearby specialist clinic and/or the distance to a secondary care hospital. These factors likely had an impact on RFEs and health problems. Second, to ensure quality, only original articles published in peer-reviewed journals were included in the present systematic review. Conference presentations and unpublished articles that were excluded from this study may contain additional research conducted in urban areas or in multiple facilities. Third, unfortunately, the type of doctors (internist/primary care physicians) and the type of training were not described in the studies included by our systematic review. Fourth, the distinction between internal medicine and non-internal medicine was not judged based on actual patient behavior under the situation that the care by various specialists was easily available. The distinction was only based on the decision by the two of the authors. In addition, whether patients with non-internal medicine related RFEs/health problems choose primary care physicians or specialists, i.e. orthopedics might depend on type of training which a physician received (internist/primary care physician). If some internists in the researches included by our systematic review acquired the knowledge and skills of primary care physician by self-directed learning, however, the proportion of non-internal medicine related RFEs and health problems might be overestimated for internists in general.

## Conclusions

In conclusion, the findings of review suggest that the ability to deal with “non-internal medicine-related” RFEs and health problems is required for primary care physicians in some rural areas of Japan. In addition, curriculum need to be combined with “non-internal medicine-related” practical training to foster aspiring generalists. We expect these findings to help facilitate improvements in the early stages of the educational system for generalists. More studies focusing on ICPC should be conducted in the future in order to better understand the current status of primary care in Japan.

Although the study was regionally limited, its result may suggest that a training system for primary care physicians to deal with a variety of RFEs and health problems is important even in countries not having gatekeeping function by primary care physician such as Japan.

## Additional file

Additional file 1:
**Table S1.** Details of included studies. (XLSX 18 kb)

## Abbreviations

ICD: The International Classification of Diseases
ICHPPC: International Classification of Health Problems in Primary Care
ICPC: International Classification of Primary Care
WONCA: The World Organization of National Colleges, Academies and Academic Associations of General Practitioners/Family Physicians,
RFEs: Reasons for encounters
